# Comprehensive analysis of life quality of patients with vitiligo in Romania: insights from a multivariate approach

**DOI:** 10.3389/fmed.2025.1613083

**Published:** 2025-05-26

**Authors:** László Fekete, Vladimir Bacârea, Gyula László Fekete, Júlia Edit Fekete, Béla Kovács

**Affiliations:** ^1^Doctoral School of Medicine and Pharmacy, George Emil Palade University of Medicine, Pharmacy, Science, and Technology of Târgu Mureș, Târgu Mureș, Romania; ^2^CMI Dermamed Private Medical Office, Târgu Mureş, Romania; ^3^Department M2, Research Methodology, George Emil Palade University of Medicine, Pharmacy, Science and Technology of Târgu Mureș, Târgu Mureş, Romania; ^4^Department M4, Dermatology, George Emil Palade University of Medicine, Pharmacy, Science and Technology of Târgu Mureș, Târgu Mureş, Romania; ^5^National Institute of Public Health, Regional Center for Public Health, Târgu Mureş, Romania; ^6^Department F1, Biochemistry and Environmental Chemistry George Emil Palade University of Medicine, Pharmacy, Science and Technology of Târgu Mureș, Târgu Mureş, Romania

**Keywords:** vitiligo, DLQI, RSE, multivariate data analysis, Romania, psychosocial burden

## Abstract

**Background:**

Vitiligo is a rare skin disorder affecting approximately 2% of the world’s population with significant impact on body image and subsequent psychosocial involvement in terms of self-esteem and quality of life. Our study focused on assessing these psychosocial aspects in Romanian vitiligo patients.

**Methods:**

Assessment of self-esteem and quality of life were evaluated by self-reported completion of the Rosenberg self-esteem and Dermatology Life Quality Index validated questionnaires. Sociodemographic data were also collected from the 114 respondents, including age, gender, residence, marital status, level of education and debut of vitiligo. Besides classical statistical evaluation, dimensionality reduction analysis techniques, such as principal component analysis and multiple correspondence analysis were employed to investigate in complexity the impact of various factors on the quality of life and self-esteem of patients with vitiligo.

**Results:**

Our findings suggest that age, marital status and disease duration were amongst the most important factors that affects the psychosocial well-being of patients with vitiligo. Elderly, married patients with long-established vitiligo scored better on the quality-of-life outcomes and higher on the self-esteem questionnaire compared to their younger, single and recently diagnosed peers with vitiligo.

**Conclusion:**

Our findings highlight the significant influence of sociodemographic factors on self-esteem and quality of life in vitiligo patients, emphasizing the need for further research and personalized support to address the psychosocial impact of the condition.

## 1 Introduction

Vitiligo is a chronic, acquired autoimmune skin disorder characterized by the absence of pigmentary cells leading to a patchy loss of skin color appearing on both visible and occult surfaces of the body. With a prevalence of 0.5–2% of the world’s population, vitiligo is considered the most frequent skin depigmentation disorder, affecting approx. 28.5 million people worldwide. Akl et al. have recently reported that the physician- or dermatologist-diagnosed lifetime prevalence of vitiligo for the general population exceeded 0.5% in countries in the Indian subcontinent and Eastern Europe with an estimate of 0.54% [0.29–1.11] in Romania (1, 2). Vitiligo can be categorized as non-segmental or generalized and segmental or localized. Non-segmental vitiligo has multifactorial etiology ranging from autoimmune disorders to various risk factors, such as genetic predisposition, familial clustering, skin cancer, or other triggers. Segmental vitiligo is presumably caused by the release of different neurochemicals by the nerve endings in the skin, destroying the melanocyte skin cells (3). Albeit, a vast treatment arsenal for vitiligo exists, including topical ointments, phototherapy, oral corticosteroids or immunosuppressive agents, surgery, or adjunctive therapies, it remains without a universal cure, remaining both a medical and humanistic burden worldwide (4, 5).

Vitiligo is known to affect patient’s quality of life characterized by psychosocial disturbances translated to both private and social interpersonal relationships (6). The recently published VALIANT study by Bibeau et al. reported a significant impact of vitiligo on quality of life and emotional well-being, particularly in patients with more than 5% of their body surface affected, Fitzpatrick skin types IV–VI, or visible skin involvement (7). Several questionnaires are available to assess the quality of life of patients with vitiligo. One of the most widely used methods is the Dermatology Life Quality Index (DLQI) questionnaire which covers the symptoms, emotions, social interactions and treatment difficulties of patients with vitiligo (8–10) or VitiQoL (Vitiligo-Specific Quality of Life Instrument) mainly developed to assess the psychosocial burden of vitiligo in terms of stigma, social anxiety and behavior (11). Regarding confidence, the Rosenberg self-esteem scale, RSE (12) or the State self-esteem scale, SSES (13) are the most widely used questionnaires focusing on self-worth and self-acceptance and temporary self-esteem fluctuations, respectively.

Our study focuses on the evaluation of life quality and self-esteem of patients with vitiligo in Central Romania based on the DLQI and RSE questionnaires. Furthermore, our goal was to elucidate complex associations between anthropometric and disease-related variables and their impact on the patient’s psychosocial well-being using dimensionality reduction statistical techniques such as principal component analysis driven hierarchical clustering analysis and multiple correspondence analysis.

## 2 Materials and methods

### 2.1 Study design

The present study was a cross-sectional, observational study based on data collection using the Dermatology Life Quality Index (DLQI) and Rosenberg Self-Esteem (RSE) questionnaires conducted between March 2022 and March 2023. The enrolled study participants were patients diagnosed with vitiligo, over 18 years of age from private outpatient offices in the region and the outpatient department of the Dermatology Clinic in Târgu Mureș. Participation in the study was voluntary, and patients were informed about the aims and scope, anonymity and compliance with the General Data Protection Regulation (GDPR). Minimal sample size was established based on obtaining pertinent insights into sociodemographic and vitiligo relationships focusing on life quality and self-esteem with a confidence level of 95% at 10% margin of error. The severity of vitiligo was classified using a simplified extent-based system as proposed by Kawakami et al. (14) which was adopted to our study design considering Grade 1 for affected skin surface below 10%, Grade 2 for 10–25%, and Grade 3 for involvement greater than 25%. The present study involving humans was approved by the Ethics Commission of the Faculty of Medicine with no. 1255/2021, respectively, the Mureș County Clinical Hospital with no. 16501/2021.

### 2.2 DLQI and RSE questionnaires

Both questionnaire surveys were conducted on their respective validated form. The questions on the DLQI questionnaire were scored on a four-point Likert scale: 0 = not at all, 1 = a little, 2 = a lot and 3 = very much. Total DLQI score was calculated by adding up the scores obtained for each question. According to these, patients were categorized in one of the following DLQI categories:

- 0–1 point: no effect on the patient’s life
- 2–5 points: small effect on the patient’s life
- 6–10 points: moderate effect on the patient’s life
- 11–20 points: very large effect on the patient’s life
- 21–30 points: extremely large effect on the patient’s life.

Furthermore, the DLQI questionnaire was evaluated based on six sub-scale established as follows: symptoms and feelings (Q1 and Q2), daily activities (Q3 and Q4), leisure (Q5 and Q6), work and school (Q7) personal relationships (Q8 and Q9) and treatment (Q10).

Similarly, the RSE questionnaire was evaluated on a four-point Guttman scale: 0 = strongly disagree, 1 = disagree, 2 = agree and 3 = strongly agree. Items 2, 5, 6, 8 and 9 were reverse scored for the evaluation. Scores between 15 and 25 were considered normal range, while scores below 15 were categorized as low self-esteem.

### 2.3 Statistical analysis

Data evaluation was carried out using GraphPad Instat® v. 3.06 (GraphPad Software Inc., El Camino Real, San Diego, USA). Two – and three–dimensional contingency tables were analyses using Fischer’s exact test and chi–square test, respectively. Respondents DLQI and RSE total scores were evaluated for normal distribution using the Kolmogorov-Smirnov test. Non–parametric tests were used to compare the total scores obtained for DLQI and RSE questionnaires – Mann–Whitney U–test for comparing two groups, and Kruskal–Wallis test or one-way ANOVA to compare three groups. Multivariate data analysis of the dataset was carried out using the SIMCA® 17 software (Sartorius Stedim Data Analytics AB, Umeå, Sweden). Principal component analysis (PCA–X) based hierarchical clustering analysis (HCA) was employed as statistical methodology to assess complex factor–to–response relationships in the presented dataset. The model was evaluated based on the goodness of fit (R^2^) and goodness of predictability (Q^2^) values. Separate designs were constructed based on respondents’ answers given to both DLQI and RSE questionnaire, as well as based a scoring system according to the guidelines given for each type of questionnaire. To validate the results obtained by categorical HCA, a correspondence analysis (CA) was carried out using the Orange® data mining software v. 3.38.1 (Bioinformatics Lab, Ljubljana, Slovenia). Statistically significant differences were considered at a cut–off of *p* < 0.05 (15).

## 3 Results

### 3.1 Anthropometric and demographic data

Anthropometric data – The questionnaires were completed by 114 persons, of which 56 male and 58 female patients, indicating an equal distribution of the respondents in terms of gender. Regarding the residence of the patients 66.6% (*n* = 76) were urban residing persons, while 33.3% (*n* = 38) were from the rural environment. The age distribution of the respondents spun between 19 and 78 years as presented in Table 1.

**Table 1:** Age distribution of the respondents.

Age distribution	Number of respondents
18–24 years	7
25–34 years	25
35–49 years	26
50–65 years	25
> 65 years	31

About two-thirds of the participants were married (64.9%, *n* = 74) and the rest had single marital status (35.1%, *n* = 40). Concerning the level of education, 79 of the respondents (69.3%) were enrolled in secondary school or graduates and 35 (30.7%) were pursuing university studies or had a BSc degree.

Data about vitiligo – more than half of the respondents had a debut of the disease over 10 years (54.4%, *n* = 62) and about a quarter between 5 and 10 years (21.9%, *n* = 25) or below 5 years (23.7%, *n* = 27). Regarding the localization of the spots, 58.8% (*n* = 67) of the cases were on visible skin surfaces, while in the rest of the cases (41.2%, *n* = 47) these were occult. Nearly half (47.4%, *n* = 54) of the respondents presented grade 1 vitiligo with an affected surface of less than 10%. Grade 2 vitiligo, with an affected skin surface between 10 and 25%, was recorded of another 40.4% (*n* = 46) of the patients. Finally, only 12.3% (*n* = 14) of the respondents presented grade 3 vitiligo, with an affected surface above 25%.

### 3.2 Evaluation of the DLQI questionnaire

**Figure 1:**
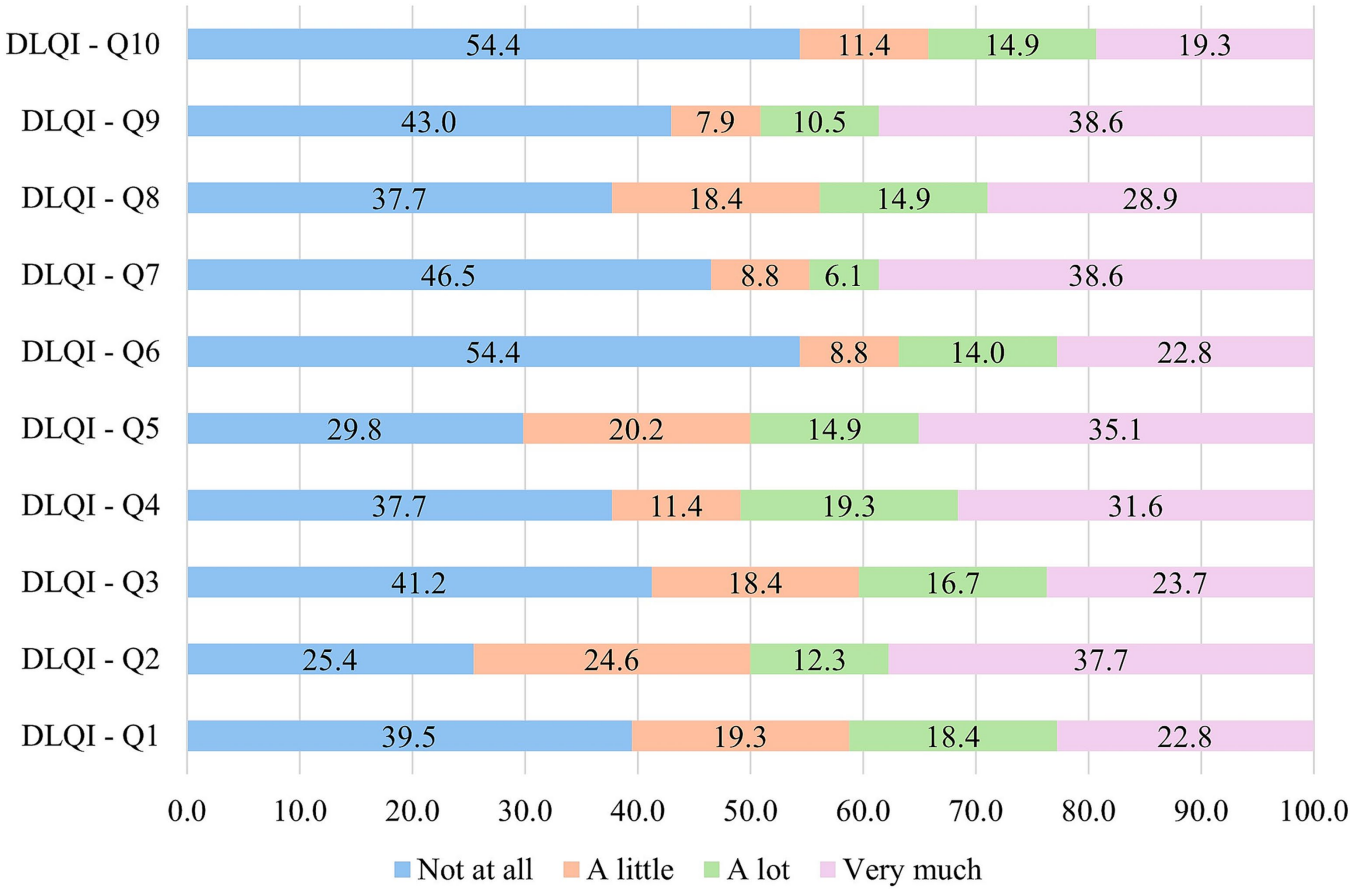
Distribution of patients’ responses to the 10 questions of the DLQI.

The frequencies of the responses given to the questions of the DLQI questionnaire are presented in Figure 1. In general, it can be stated that no statistically significant differences were obtained between the anthropometric variables and vitiligo based on the total DLQI scores obtained by the respondents. However, it is worth noting that according to the marital status of the respondents the differences are only slightly superior to the cut–off limit of *p*. The statistical results for total DLQI score are presented in Table 2, while the results obtained for each question are included in Supplementary Table 1.

**Table 2:** Statistical results of the total DLQI scores based on respondents anthropometric variables.

Anthropometric variables	Statistical analysis of total DLQI scores									
	Category	*N*	Average	Min	Q1	Median	Q3	Max	*IQR*	*p*
Age	18–40 y	40	15.0	0	5.25	18	24	29	18.75	0.159*^a^*
	41–60 y	38	13.7	0	2.5	15.5	22	28	19.5	
	> 60 y	36	11.0	0	3	10	16.5	30	13.5	
Residence	Urban	76	13.5	0	3.75	14.5	21.25	30	17.5	0.921*^b^*
	Rural	38	13.0	0	3	11.5	22	29	19	
Gender	M	56	12.6	0	2.75	10.5	21.25	28	18.5	0.504*^b^*
	F	58	14.0	0	6	15	22	30	16	
Localisation	Occult	47	13.6	0	4.5	15	21.5	30	17	0.738*^b^*
	Visible	67	13.1	0	2.5	14	22	28	19.5	
Marital status	Married	74	12.1	0	3	11.5	21	30	18	0.055*^b^*
	Single	40	15.6	0	5.25	18	24	29	18.75	
Debut	< 5 y	27	14.7	0	3.5	18	24	27	20.5	0.452*^b^* 0.210*^a, c^*
	5–10 y	25	14.6	0	3	18	22	27	19	
	> 10 y	62	12.2	0	3	12	19.5	30	16.5	
Level of education	Secondary school	79	13.2	0	3	14	22	30	19	0.859*^b^*
	BSc	35	13.6	0	2.5	16	22.5	28	20	
Affected surface	Grade 1	54	13.5	0	2	15.5	21.75	29	19.75	0.835*^a^*
	Grade 2	46	12.8	0	3	11.5	22	30	19	
	Grade 3	14	14.3	1	7	14.0	22	28	15	

^a^Kruskal–Wallis; ^b^Mann–Whitney U-test; ^c^debut < 10 y vs. > 10 y; N, no. of respondents; Q1, first quartile; Q3, third quartile; IQR, interquartile range.

Symptoms and feelings (DLQI Q1 and Q2) – 58.8% of the respondents reported no (39.5%, *n* = 45) or little (19.3%, *n* = 22) skin affection in terms of pruritus or affliction (Q1). In contrast, 41.2% of the people noted that these symptoms were existent at the moment of evaluation with 26 persons reporting appreciable skin irritation due to the presence of vitiligo. Statistically notable differences were reported based on the debut of the disease (*p* = 0.0014), where people with vitiligo above 10 years reported less symptoms compared to those with less than 10 years from the debut of vitiligo. Furthermore, statistically significant differences were obtained according to gender (*p* = 0.029), however considering only binary outcomes no differences were obtained based on Fischer’s exact test (*p* = 0.707). Concerning embarrassment or self–consciousness no statistically significant differences were observed based on the evaluation of any other anthropometric data (Q2).

Daily activities (DLQI Q3 and Q4) – concerning routine daily activities significant differences were observed in an age dependent manner regarding the public appearance of the respondents, e.g., shopping (*p* = 0.032). Elderly patients noted less concern regarding the social judgment compared to young adults and adults between 41–60 years. Despite this observation, no relevant differences were identified concerning the clothing habits between ages groups (*p* = 0.850). The other anthropometric data did not show noticeable differences between the answers given to Q3 and Q4.

Leisure (DLQI Q5 and Q6) and work and school (DLQI Q7) – no statistically significant differences were observed between any of the anthropometric data analyzed.

Personal relationships (DLQI Q8 and Q9) – personal relationships (Q8) were significantly affected by the martial status of the respondents (*p* = 0.038), where 50% of the single persons have reported that vitiligo affects their personal relationships, compared to 40% of the married respondents. From another perspective, if only binary outcomes were evaluated no statistically significant differences were observed according to the marital status (*p* = 0.429). Difficulties concerning sexual life were reported mainly by respondents below the age of 60 years, showing noticeable differences between the age groups (*p* = 0.048). Similarly, based on the localization of vitiligo significant differences were observed between the visible and occult group (*p* = 0.044), however once again, if binary outcomes are considered the differences were not considerable (*p* = 0.452).

Treatment (DLQI Q10) – regarding the time consumption expended on treatment significant differences were obtained for the affected surface area of the skin. Persons with grade 1 vitiligo generally reported no affection in terms of treatment demands compared to grade 2 (*p* = 0.016) or grade 2/3 patients (*p* = 0.015).

### 3.3 Evaluation of the RSE questionnaire

**Figure 2:**
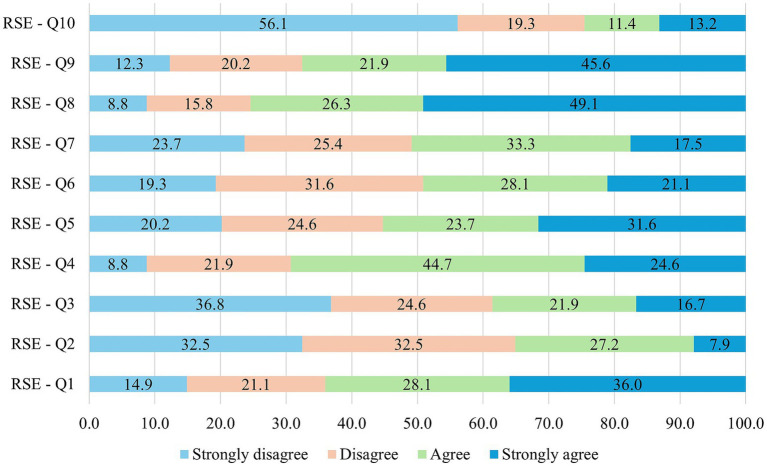
Distribution of patients’ responses to the 10 questions of the RSE questionnaire.

The frequency of responses given to the questions in the RSE questionnaire are presented in Figure 2. In terms of RSE total score, low self-esteem was considerably higher in an age (*χ*^2^ (2, 114) = 11.980, *p* = 0.003) and marital status (*χ*^2^ (1, 114) = 4.580, *p* = 0.032) dependent manner. Considering the total RSE score albeit that according to age no statistically significant differences were obtained (*p* = 0.071), based on different age categories a notable difference was observed between patients between 18 and 40 years of age compared to elderly patients above 60 years of age (*p* = 0.026). The statistical results for total RSE score are presented in Table 3.

**Table 3:** Statistical results of the total RSE scores based on respondents anthropometric variables.

Anthropometric variables	Statistical analysis of total RSE scores									
	Category	*N*	Average	Min	Q1	Median	Q3	Max	IQR	*p*
Age	18–40 y	40	12.4	3	8.5	11	17.25	29	8.75	0.071*^a^*
	41–60 y	38	13.6	3	8	12.5	18	30	10	
	> 60 y	36	15.2	7	11.5	14.5	18	27	6.5	
Residence	Urban	76	13.7	3	9	13	18	30	9	0.947*^b^*
	Rural	38	13.6	3	9	13.5	17.5	27	8.5	
Gender	M	56	14.2	3	9.75	13	18.5	30	8.75	0.540*^b^*
	F	58	13.2	3	9	13	18	27	9	
Localisation	Occult	47	14.3	3	9	14	18.5	30	9.5	0.432*^b^*
	Visible	67	13.3	3	9	13	18	29	9	
Marital status	Married	74	14.3	3	10	14	18	30	8	0.076*^b^*
	Single	40	12.6	3	6.75	10.5	19	29	12.25	
Debut	< 5 y	27	13.3	3	8	11	19	29	11	0.547*^c^* 0.132*^d^*
	5–10 y	25	12.7	3	8	12	18	27	10	
	> 10 y	62	14.3	3	10	14	18	30	8	
Level of education	Secondary school	79	13.7	3	9	13	18	30	9	0.917*^b^*
	BSc	35	13.7	3	9	13	18.5	29	9.5	
Affected surface	Grade 1	54	13.1	3	9	12	17.75	29	8.75	0.625*^c^*
	Grade 2	46	14.2	3	9	13	18	30	9	
	Grade 3	14	14.6	4	12	15	19.5	24	7.5	

^a^Kruskal–Wallis; ^b^Mann–Whitney U-test; ^c^One-way ANOVA; ^d^debut < 10 y vs. > 10 y; N; no. of respondents; Q1, first quartile; Q3, third quartile; IQR, interquartile range.

### 3.4 Exploratory data analysis

Based on the evaluated data and by considering the psychological complexity of skin related diseases, i.e., vitiligo, PCA based HCA and CA were employed to investigate and describe underlying relationships between anthropometric data, vitiligo and life quality and self-esteem.

**Figure 3:**
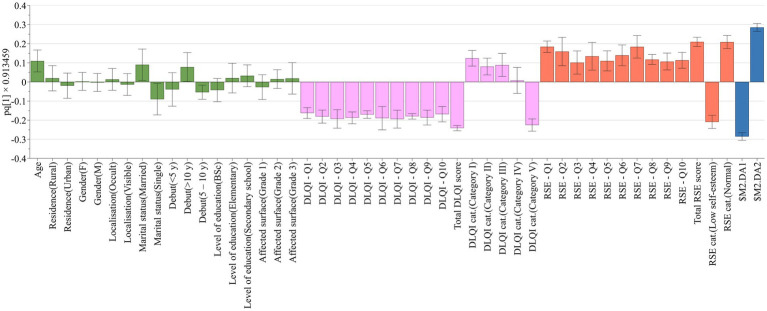
Life quality and self-esteem differences according to sociodemographic clustering of patients. Anthropometric and disease related data are shown with green bars, DLQI questions and categories with lavender bars, RSE questions and categories with brownish-red bars, while clusters with blue bars.

Multivariate data analysis – the HCA analysis based on numerical data revealed a clustering of the patients on the first principal component. The first group of patients comprised of single, 18–40 years respondents with a debut of vitiligo of less than 10 years. Conversely, the second group contained married, elderly patients with more than 10 years of vitiligo. No other sociodemographic differences were identified between the resulting clusters. The results obtained revealed important differences between the two clusters in terms of responses given to both the DLQI and RSE questionnaires. Young, single patients tend to give higher scores to all questions regarding the life quality questionnaire, indicating that vitiligo affects a lot or very much their symptoms, daily activities, leisure or work demands, personal relationships or treatment bearings, being generally classified into category V indicating that vitiligo has an extremely large effect on patients’ life. In contrast, elderly, married patients are less affected by the presence of vitiligo and no-to-moderate (cat. I – III.) effect on the patient’s life was observed as the responses given to DLQI Q1–Q10 are mainly “not at all” (Figure 3). The correlation matrix between DLQI and RSE questions is presented in Supplementary Table 3.

**Figure 4:**
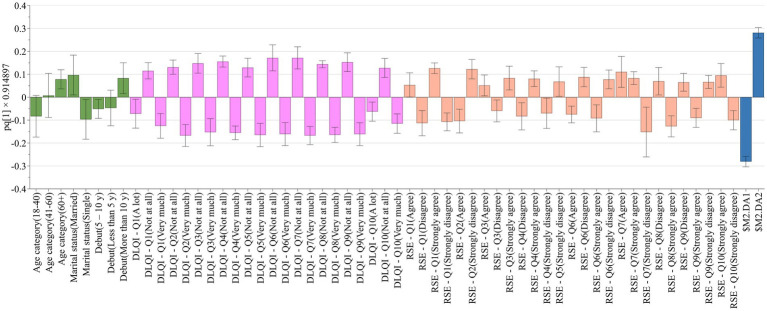
Differences in responses given to the DLQI and RSE questionnaires according to sociodemographic clustering of patients. Anthropometric and disease related data are shown with green bars, DLQI questions and categories with lavender bars, RSE questions and categories with brownish-red bars, while clusters with blue bars.

By analyzing the categorical dataset based on the previous clustering of the patients it was observed that indeed elderly, married patients with long–standing vitiligo generally responded with “not at all” to the questions of the DLQI questionnaire, compared to the younger people with responses ranging from “a lot” to “very much.” Concurrently, the affected life quality of younger respondents is also reflected in the responses given to the RSE questionnaire (Figure 4).

**Figure 5:**
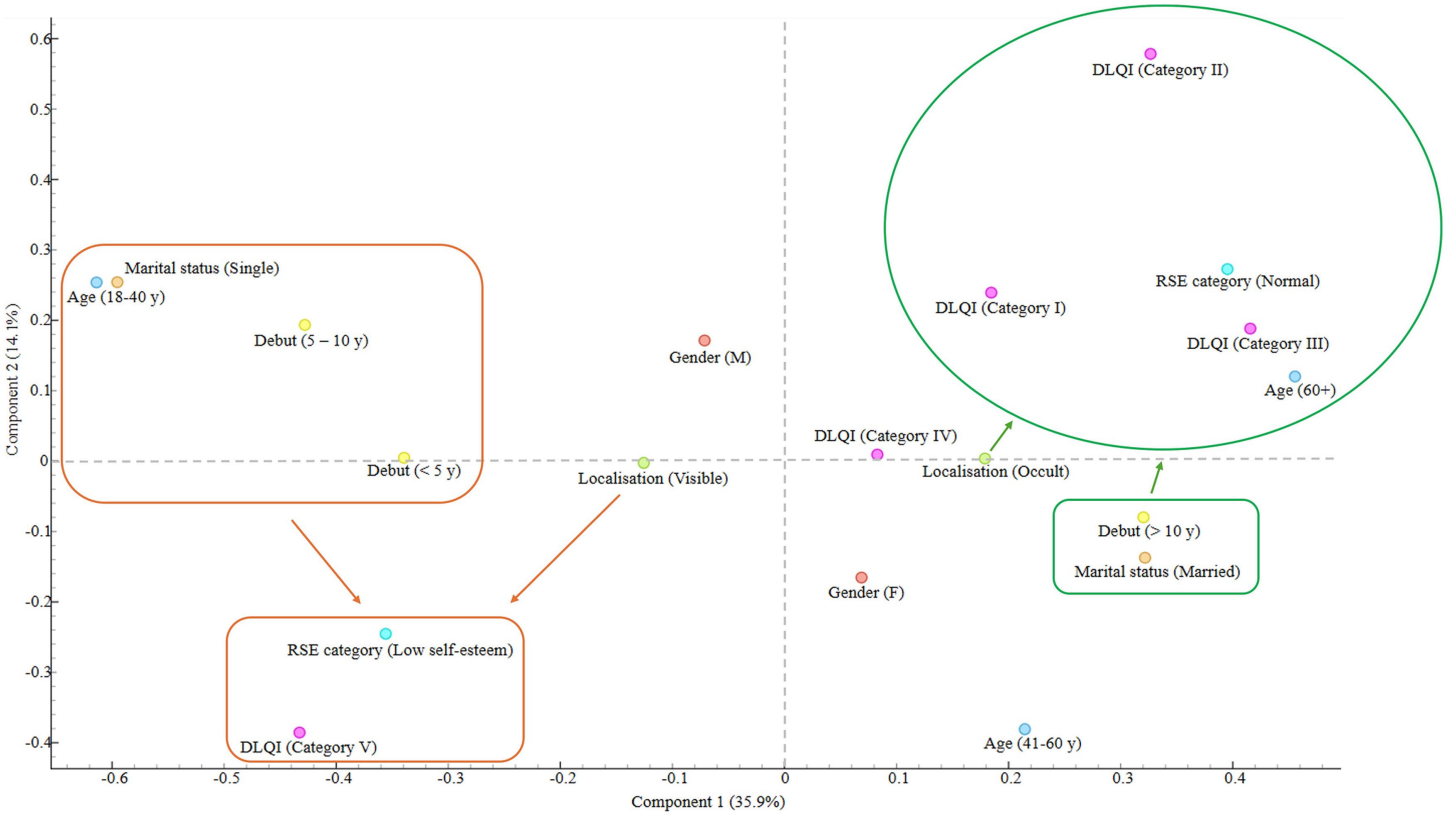
Distribution and clustering of data based on multiple correspondence analysis.

The results obtained by PCA were underpinned by evaluation of the dataset using MCA (Figure 5). Similar data clustering can be observed according to the four quadrants of the cartesian plane. The first quadrant comprises of the elderly patients with DLQI categories of I – III, showing normal self–esteem according to the evaluation of the RSE questionnaire. This is complemented by the variables existent in the fourth quadrant, where married people with long–lasting vitiligo, mainly present on occult surfaces are showing an inclusion tendency toward this group. On the other pole, quadrants two and three were represented by young, single patients generally being classified as category V outcomes according to the DLQI questionnaire, and presenting low self-esteem. Visible localisation of vitiligo and men usually present a tendecy toward these obsevations.

**Figure 6:**
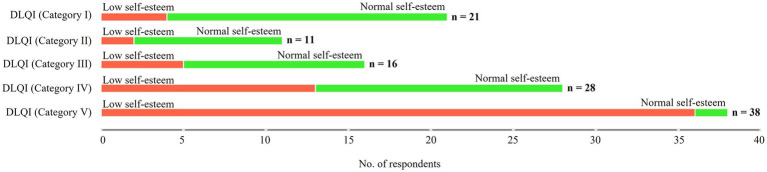
Interrelation between DLQI and RSE categories of patients with vitiligo.

Based on DLQI and RSE categories statistically significant differences were obtained between DLQI categories and corresponding RSE classifications, *χ*^2^ (4, 114) = 45.13, *p* < 0.001. DLQI category I and II patients were generally categorized as having normal self-esteem, however higher categories gradually tend to show psychological distress, reflecting in lower self-esteem (Figure 6).

Table 4 summarizes the correlation between the DLQI and RSE categorization of the respondents according to the HCA conducted on numerical data. The results indicated that indeed normal self-esteem was correlated with lower impact of vitiligo on the quality of life of the patients, but this tendency gradually switched to a significantly negative correlation in the case of DLQI category V respondents.

**Table 4:** Correlation matrix between DLQI and RSE categories.

	RSE – Low-self esteem	RSE – Normal self-esteem
DLQI – Category I	−0.32	0.32
DLQI – Category II	−0.23	0.23
DLQI – Category III	−0.17	0.17
DLQI – Category IV	−0.071	0.071
DLQI – Category V	0.6	−0.6

## 4 Discussion

Vitiligo is a skin disorder that significantly affects an individual’s psychosocial well-being and perceived quality of life, often leading to implications for self-esteem among those affected. Furthermore, every patient’s experience is distinct, shaped by differences in gender, cultural background, and the healthcare system they navigate, which further underpins the needs to assess the quality of life of patients with vitiligo and to understand patients coping strategies with the burden of the disease (16).

### 4.1 Life quality and self-esteem of patients with vitiligo

The DLQI serves as a crucial tool in evaluating the quality of life (QoL) among dermatology patients, including those with vitiligo. Several studies have demonstrated that vitiligo can result in marked impairments in quality of life, indicating not only the visible impact of skin lesions but also internal struggles related to self-worth. In our study the average DLQI score in our cohort was 13.3 which is considerably higher than the results presented by other research groups, of 6–7 (17, 18). In an opinion published by Chernyshov et al. it was disclosed that patients with darker skin types reported higher DLQI scores than those with fairer skin types (19). Although, the 114 patients enrolled in our study were all Caucasian participants with phototypes between I and III on the Fitzpatrick skin type scale, somewhat limiting our comparative analysis with other global studies, it is worth noting that compared to other European countries our results indicate the highest DLQI scores hinting a moderate impact on patients’ life. However, considering the Balkan region our results are comparable with those obtained by Teovska-Mitreska in North-Macedonian patients with and average DLQI score of 11.7 (20). This observation may also be associated with stigma or misconceptions surrounding vitiligo in traditional societies, including Romania. In such regions, this can lead to social isolation, avoidance of relationships and discrimination on work or school settings. QoL is also affected by the own perception made by patients suffering from this disease. Psychometric analysis using the RSE questionnaire have revealed a considerable impact on the self-esteem of patients suffering from vitiligo in terms of body image on a neurobiological plain based on demographic characteristics and disease features (21, 22). In our study the average RSE score was 13.7, suggesting somewhat a lower-than-normal self-esteem amongst the enrolled patients.

Life quality among individuals with vitiligo is notably affected by demographic factors such as age, gender, and marital status. Studies show that these variables are closely linked to the psychological and social challenges associated with the condition, which in turn shape how patients perceive their overall well-being. The mean age of the patients enrolled in our study was 49.1 years, evenly distributed among the presented age categories. Researchers consistently show that younger patients score higher on the DLQI questionnaire and experience greater emotional and social distress than their elderly peers (23, 24). Despite that in our study no statistically significant differences were obtained in an age dependent manner, decremental values of total DLQI scores were observed according to age categories, which underpins the observation that life quality can be perceived differently by adult and elderly patients with vitiligo. This observation is also supported by the gradually increasing RSE scores according to age categories, where elderly patients score above 15 on average, suggesting a rather normal self-esteem compared to young respondents reaching the lowest average value of 12.4 considering all sociodemographic variables. Referring to gender, usually female patients tend to experience more significant impact on the quality of life than males, mainly driven by sociodemographic variables (25, 26). In our cohort, there was no statistically significant difference in mean DLQI score between male and female vitiligo patients, however in-line with the literature data female patients scored higher than their male counterparts and this is also evidenced by the lower RSE scores for female patients. It is generally acknowledged, that patients with skin affection, like vitiligo, may experience reticence in intimacy and sexual disfunction (27). While some studies report a higher prevalence of vitiligo among women, inconsistent findings may stem from varying assessment methods and cultural differences, with individuals – particularly those with low self-esteem – being more prone to experiencing sexual difficulties (28). Our findings indicate a trend toward statistical significance based on marital status when considering both the DLQI and RSE scores, which may help explain the inconsistencies reported in the literature.

No noticeable differences were obtained according to education, localized and generalized, stable and unstable or with or without facial involvement amongst vitiligo patients. These findings are consistent with those presented in the literature. According to our results, the duration of the disease (i.e., the age of onset: infancy/childhood vs. adolescence/adult age) was not statistically related to the mean DLQI score. Among the studies which analysed this variable, only the results published by Parsad et al. (29) showed differences in DLQI score according to the length of the disease, however on the contrary, other authors are in agreement with our findings (30). When DLQI score was considered with respect to the visibility of the affected sites, e.g., visible and occult areas, the observations of the published studies are inconsistent. According to Ongenae et al. the overall DLQI score correlated with the localization to face, trunk and feet, but did not correlate with localization to hands (31). Conversely, the visibility of sites involved, grouped together, significantly correlated with higher DLQI (32). Additionally, Belhadjali et al. did not observe any significant difference in DLQI score between patients with vitiligo involving covered and uncovered skin, nor in patients in which face and genitalia were, respectively, involved/uninvolved (30).

Concerning the affected skin surface no statistical differences were noted between grade 1, 2 and 3 patients. However, it is worth noting that respondents with grade 3 vitiligo scored higher on both the DLQI and RSE questionnaires than their peers with less affected skin surface. Morales-Sánchez et al. (33) have also reported that the skin surface affected did not show a noticeable impact on the quality of life, as similarly assessed by the DLQI questionnaire. Recent clinical trials have reported that the reduced quality of life expressed by individuals with long-standing vitiligo is likely attributable to the chronic, relapsing–remitting nature of the condition and difficulties in maintaining treatment adherence (34). This trend was also evident in our study, as reflected in the responses to DLQI question 10, where patients with grade 2 and grade 3 vitiligo reported greater impact related to treatment options compared to those with less extensive disease involvement. This may be further influenced by cultural factors – particularly in more traditional societies such as Romania – where reliance on indigenous medical practices and widespread beliefs about the incurability of vitiligo are persisting.

### 4.2 Multivariate data analysis on the quality of life and self-esteem of patients with vitiligo

Studies have shown that individuals with vitiligo frequently experience a diminished quality of life due to social stigma and the visible nature of the condition, which can lead to psychological distress. For instance, Alfahl et al. found that most vitiligo patients reported low self-esteem, underscoring the emotional ramifications of the disease (35). Additionally, Bonotis et al. highlighted that self-esteem, perceived disease severity, and personality traits such as neuroticism are significant predictors of health-related quality of life (HRQoL) impairment in vitiligo patients (22). These findings suggest that the psychological burden of living with vitiligo is closely intertwined with self-esteem and general quality of life perceptions. Our findings further indicate a potential association between vitiligo, diminished quality of life, and lower self-esteem; however, this relationship warrants a more nuanced and comprehensive analysis beyond basic pairwise comparisons. Using PCA and MCA it was shown that a combination of sociodemographic and clinical factors may jointly lead to different valuations of self-perception and QoL among patients with vitiligo. Habituation to vitiligo over time, due to ageing or disease progression as well as fulfillment by partner acceptance can lead to a higher perception of life quality and higher self-esteem. On the contrary, challenges in young adulthood seeking social integration and forming intimate relationships coupled with newly acquired skin changes may negatively affect perceptions of life quality. This might be driven by low self-esteem, rooted in stigma and misjudgement, especially in more traditional societies.

### 4.3 Strengths and limitations of the study and future perspectives

As to our knowledge this is the first paper evaluating the quality of life and the burden of the disease in Romanian vitiligo patients using the DLQI and RSE questionnaires. Using different dimensionality reduction analysis tools, we could explore in greater depth the impact of sociodemographic variables on the quality of life and self-esteem of patients with vitiligo. It is also recognized that self-report questionnaires are susceptible to response bias, and that self-esteem and quality of life may be either under- or overestimated due to factors beyond the disease itself. Furthermore, larger studies conducted on population-based samples are needed to confirm or infirm our findings, possibly involving primary care physicians. Also, it is recommended that the assessment of quality of life in patients with vitiligo be complemented by additional validated psychometric instruments, such as the Beck Depression Inventory for evaluating depressive symptoms and the State Anxiety Inventory-1 (STAI-1) for measuring state anxiety and FSFI (Female Sexual Function Index Scoring) and/or IIEF (International Index of Erectile Function) to address intimacy hurdles among patients with vitiligo.

## 5 Conclusion

In summary, our findings indicate that the quality of life and self-esteem of patients with vitiligo is crucially impacted according to sociodemographic variables. Although pairwise comparisons of the DLQI and RSE total scores did not show statistically significant differences, principal component analysis and multiple correlation analysis revealed meaningful underlying patterns. Variables such as age, marital status and disease duration emerged as key factors differentiating responses on both the DLQI and RSE scales. Generally, elderly, married patients with longstanding vitiligo have reported higher self-confidence and a lesser impact to their quality of life compared to their younger, single and newly diagnosed peers.

In addition, it is essential to conduct further research on the quality of life in vitiligo patients. Providing personalized medical and psychological support are crucial in improving patient care and addressing the psychosocial burden of the disease. Ultimately, improving patients’ subjective appraisal of their condition is essential to better cope with the emotional and social impact of the condition.

## Data Availability

The original contributions presented in the study are included in the article/Supplementary material, further inquiries can be directed to the corresponding authors.
