# Supplementary material for: Comprehensive analysis of life quality of patients with vitiligo in Romania: insights from a multivariate approach
Source: Front Med (Lausanne). 2025 May 26;12:1613083. doi: 10.3389/fmed.2025.1613083 (PMC12146156; doi:10.3389/fmed.2025.1613083)
Supplement: Supplementary file 1 [file Table_1.docx]

**Supplementary Table 1.** Statistical analysis of the responses given by the respondents to the items of DLQI questionnaire based on anthropometric variables

| **Anthropometric category** | **Subgroup** | **Not at all** | **A little** | **A lot** | **Very much** | **χ^2^ - DLQI Q1 - Skin affection** |
| --- | --- | --- | --- | --- | --- | --- |
| Age | 18-40 | 13 | 6 | 10 | 11 | χ^2^ (114, 6) = 5.789, *p* = 0.447 |
|  | 41-60 | 15 | 10 | 7 | 6 |  |
|  | >60 | 17 | 6 | 4 | 9 |  |
| Residence | Urban | 28 | 16 | 15 | 17 | χ^2^ (114, 3) = 0.9972, *p* = 0.802 |
|  | Rural | 17 | 6 | 6 | 9 |  |
| Gender | M | 28 | 6 | 12 | 10 | **χ^2^ (114, 3) = 9.015, *p* = 0.029** Fischer's exact test, *p* = 0.707 |
|  | F | 17 | 16 | 9 | 16 |  |
| Localisation | Visible | 27 | 14 | 8 | 18 | χ^2^ (114, 3) = 5.122, *p* = 0.163 |
|  | Occult | 18 | 8 | 13 | 8 |  |
| Marital status | Single | 11 | 7 | 10 | 12 | χ^2^ (114, 3) = 5.187, *p* = 0.159 |
|  | Married | 34 | 15 | 11 | 14 |  |
| Debut | < 5y | 11 | 2 | 5 | 9 | **χ^2^ (114, 3) = 15.620, p = 0.0014**, *less than 10 y vs. more than 10 y* Fischer's exact test, *p* = 0.0013 |
|  | 5-10 y | 8 | 1 | 8 | 8 |  |
|  | >10 y | 26 | 19 | 8 | 9 |  |
| Level of education | Secondary school | 30 | 14 | 14 | 21 | χ^2^ (114, 3) = 2.154, *p* = 0.541 |
|  | BSc | 15 | 8 | 7 | 5 |  |
| Affected surface | Grade 1 | 26 | 7 | 8 | 13 | χ^2^ (100, 3) = 4.744, *p* = 0.192, *Grade 1 vs. Grade 2* χ^2^ (114, 3) = 4.744, *p* = 0.180*, Grade 1 vs. Grade 2 and 3* |
|  | Grade 2 | 14 | 11 | 11 | 10 |  |
|  | Grade 3 | 5 | 4 | 2 | 3 |  |
| **Anthropometric category** | **Subgroup** | **Not at all** | **A little** | **A lot** | **Very much** | **χ^2^ - DLQI Q2 - Embarrassment or self–consciousness** |
| Age | 18-40 | 7 | 14 | 3 | 16 | χ^2^ (114, 6) = 7.151, *p* = 0.307 |
|  | 41-60 | 10 | 6 | 7 | 15 |  |
|  | >60 | 12 | 8 | 4 | 12 |  |
| Residence | Urban | 17 | 21 | 10 | 28 | χ^2^ (114, 3) = 1.909, *p* = 0.592 |
|  | Rural | 12 | 7 | 4 | 15 |  |
| Gender | M | 16 | 16 | 6 | 18 | χ^2^ (114, 3) = 2.273, *p* = 0.518 |
|  | F | 13 | 12 | 8 | 25 |  |
| Localisation | Visible | 17 | 19 | 8 | 23 | χ^2^ (114, 3) = 1.465, *p* = 0.690 |
|  | Occult | 12 | 9 | 6 | 20 |  |
| Marital status | Single | 5 | 14 | 4 | 17 | χ^2^ (114, 3) = 7.423, *p* = 0.060 |
|  | Married | 24 | 14 | 10 | 26 |  |
| Debut | < 5y | 7 | 6 | 2 | 12 | χ^2^ (114, 6) = 5.012, *p* = 0.542 χ^2^ (114, 3) = 3.293, *p* = 0.349, *less than 10 y vs. more than 10 y* |
|  | 5-10 y | 3 | 8 | 3 | 11 |  |
|  | >10 y | 19 | 14 | 9 | 20 |  |
| Level of education | Secondary school | 19 | 19 | 9 | 32 | χ^2^ (114, 3) = 0.917, *p* = 0.821 |
|  | BSc | 10 | 9 | 5 | 11 |  |
| Affected surface | Grade 1 | 13 | 14 | 5 | 22 | χ^2^ (100, 3) = 1.430, *p* = 0.698, *Grade 1 vs. Grade 2* χ^2^ (114, 3) = 1.164, *p* = 0.762*, Grade 1 vs. Grade 2 and 3* |
|  | Grade 2 | 13 | 9 | 7 | 17 |  |
|  | Grade 3 | 3 | 5 | 2 | 4 |  |

| **Anthropometric category** | **Subgroup** | **Not at all** | **A little** | **A lot** | **Very much** | **χ^2^ - DLQI Q3 – Daily activities (shopping)** |
| --- | --- | --- | --- | --- | --- | --- |
| Age | 18-40 | 15 | 10 | 5 | 10 | **χ^2^ (114, 6) = 13.829, *p* = 0.032** |
|  | 41-60 | 13 | 3 | 12 | 10 |  |
|  | >60 | 19 | 8 | 2 | 7 |  |
| Residence | Urban | 29 | 18 | 11 | 18 | χ^2^ (114, 3) = 4.608, *p* = 0.201 |
|  | Rural | 18 | 3 | 8 | 9 |  |
| Gender | M | 26 | 8 | 12 | 10 | χ^2^ (114, 3) = 4.819, *p* = 0.186 |
|  | F | 21 | 13 | 7 | 17 |  |
| Localisation | Visible | 28 | 13 | 10 | 16 | χ^2^ (114, 3) = 0.395, *p* = 0.941 |
|  | Occult | 19 | 8 | 9 | 11 |  |
| Marital status | Single | 14 | 9 | 6 | 11 | χ^2^ (114, 3) = 1.618, *p* = 0.655 |
|  | Married | 33 | 12 | 13 | 16 |  |
| Debut | < 5y | 11 | 2 | 4 | 10 | χ^2^ (114, 6) = 5.209, *p* = 0.517 χ^2^ (114, 3) = 1.665, *p* = 0.648, *less than 10 y vs. more than 10 y* |
|  | 5-10 y | 10 | 6 | 4 | 5 |  |
|  | >10 y | 26 | 13 | 11 | 12 |  |
| Level of education | Secondary school | 35 | 13 | 13 | 18 | χ^2^ (114, 3) = 1.225, *p* = 0.747 |
|  | BSc | 12 | 8 | 6 | 9 |  |
| Affected surface | Grade 1 | 24 | 10 | 7 | 13 | χ^2^ (100, 3) = 0.917, *p* = 0.821, *Grade 1 vs. Grade 2* χ^2^ (114, 3) = 1.109, *p* = 0.775*, Grade 1 vs. Grade 2 and 3* |
|  | Grade 2 | 18 | 9 | 9 | 10 |  |
|  | Grade 3 | 5 | 2 | 3 | 4 |  |
| **Anthropometric category** | **Subgroup** | **Not at all** | **A little** | **A lot** | **Very much** | **χ^2^ - DLQI Q4 – Clothing** |
| Age | 18-40 | 12 | 4 | 9 | 15 | χ^2^ (114, 6) = 2.657, *p* = 0.850 |
|  | 41-60 | 16 | 4 | 6 | 12 |  |
|  | >60 | 15 | 5 | 7 | 9 |  |
| Residence | Urban | 28 | 7 | 16 | 25 | χ^2^ (114, 3) = 1.497, *p* = 0.683 |
|  | Rural | 15 | 6 | 6 | 11 |  |
| Gender | M | 25 | 6 | 9 | 16 | χ^2^ (114, 3) = 2.354, *p* = 0.502 |
|  | F | 18 | 7 | 13 | 20 |  |
| Localisation | Visible | 23 | 9 | 15 | 20 | χ^2^ (114, 3) = 2.040, *p* = 0.564 |
|  | Occult | 20 | 4 | 7 | 16 |  |
| Marital status | Single | 11 | 4 | 10 | 15 | χ^2^ (114, 3) = 3.535, *p* = 0.316 |
|  | Married | 32 | 9 | 12 | 21 |  |
| Debut | < 5y | 9 | 2 | 5 | 11 | χ^2^ (114, 6) = 3.768, *p* = 0.708 χ^2^ (114, 3) = 1.275, *p* = 0.735, *less than 10 y vs. more than 10 y* |
|  | 5-10 y | 8 | 5 | 5 | 7 |  |
|  | >10 y | 26 | 6 | 12 | 18 |  |
| Level of education | Secondary school | 29 | 9 | 15 | 26 | χ^2^ (114, 6) = 0.2272, *p* = 0.973 |
|  | BSc | 14 | 4 | 7 | 10 |  |
| Affected surface | Grade 1 | 20 | 7 | 9 | 18 | χ^2^ (100, 3) = 0.9779, *p* = 0.807, *Grade 1 vs. Grade 2* χ^2^ (114, 3) = 0.6696, *p* = 0.873*, Grade 1 vs. Grade 2 and 3* |
|  | Grade 2 | 19 | 5 | 10 | 12 |  |
|  | Grade 3 | 4 | 1 | 3 | 6 |  |

| **Anthropometric category** | **Subgroup** | **Not at all** | **A little** | **A lot** | **Very much** | **χ^2^ - DLQI Q5 – Social or leisure activities** |
| --- | --- | --- | --- | --- | --- | --- |
| Age | 18-40 | 10 | 9 | 5 | 16 | χ^2^ (114, 6) = 4.138, *p* = 0.658 |
|  | 41-60 | 12 | 7 | 4 | 15 |  |
|  | >60 | 12 | 7 | 8 | 9 |  |
| Residence | Urban | 24 | 17 | 9 | 26 | χ^2^ (114, 3) = 2.270, *p* = 0.518 |
|  | Rural | 10 | 6 | 8 | 14 |  |
| Gender | M | 18 | 10 | 4 | 24 | χ^2^ (114, 3) = 6.841, *p* = 0.077 |
|  | F | 16 | 13 | 13 | 16 |  |
| Localisation | Visible | 23 | 14 | 12 | 18 | χ^2^ (114, 3) = 5.258, *p* = 0.154 |
|  | Occult | 11 | 9 | 5 | 22 |  |
| Marital status | Single | 13 | 6 | 5 | 16 | χ^2^ (114, 3) = 1.630, *p* = 0.653 |
|  | Married | 21 | 17 | 12 | 24 |  |
| Debut | < 5y | 7 | 3 | 4 | 13 | χ^2^ (114, 6) = 3.958, *p* = 0.682 χ^2^ (114, 3) = 2.674, *p* = 0.448, *less than 10 y vs. more than 10 y* |
|  | 5-10 y | 8 | 5 | 3 | 9 |  |
|  | >10 y | 19 | 15 | 10 | 18 |  |
| Level of education | Secondary school | 23 | 13 | 16 | 27 | χ^2^ (114, 3) = 6.791, *p* = 0.079 |
|  | BSc | 11 | 10 | 1 | 13 |  |
| Affected surface | Grade 1 | 16 | 11 | 9 | 18 | χ^2^ (100, 3) = 0.314, *p* = 0.957, *Grade 1 vs. Grade 2* χ^2^ (114, 3) = 0.305, *p* = 0.959*, Grade 1 vs. Grade 2 and 3* |
|  | Grade 2 | 15 | 10 | 6 | 15 |  |
|  | Grade 3 | 3 | 2 | 2 | 7 |  |
| **Anthropometric category** | **Subgroup** | **Not at all** | **A little** | **A lot** | **Very much** | **χ^2^ - DLQI Q6 – Physical activities** |
| Age | 18-40 | 19 | 4 | 6 | 11 | χ^2^ (114, 6) = 5.606, *p* = 0.469 |
|  | 41-60 | 21 | 1 | 6 | 10 |  |
|  | >60 | 22 | 5 | 4 | 5 |  |
| Residence | Urban | 43 | 5 | 11 | 17 | χ^2^ (114, 3) = 1.502, *p* = 0.682 |
|  | Rural | 19 | 5 | 5 | 9 |  |
| Gender | M | 30 | 2 | 10 | 14 | χ^2^ (114, 3) = 4.785, *p* = 0.188 |
|  | F | 32 | 8 | 6 | 12 |  |
| Localisation | Visible | 38 | 6 | 11 | 12 | χ^2^ (114, 3) = 2.534, *p* = 0.469 |
|  | Occult | 24 | 4 | 5 | 14 |  |
| Marital status | Single | 18 | 3 | 5 | 14 | χ^2^ (114, 3) = 5.232, *p* = 0.156 |
|  | Married | 44 | 7 | 11 | 12 |  |
| Debut | < 5y | 15 | 3 | 2 | 7 | χ^2^ (114, 6) = 3.101, *p* = 0.796 χ^2^ (114, 3) = 1.148, *p* = 0.765, *less than 10 y vs. more than 10 y* |
|  | 5-10 y | 11 | 2 | 5 | 7 |  |
|  | >10 y | 36 | 5 | 9 | 12 |  |
| Level of education | Secondary school | 43 | 6 | 9 | 21 | χ^2^ (114, 3) = 3.295, *p* = 0.348 |
|  | BSc | 19 | 4 | 7 | 5 |  |
| Affected surface | Grade 1 | 26 | 6 | 9 | 13 | χ^2^ (100, 3) = 1.981, *p* = 0.576, *Grade 1 vs. Grade 2* χ^2^ (114, 3) = 1.953, *p* = 0.582*, Grade 1 vs. Grade 2 and 3* |
|  | Grade 2 | 28 | 3 | 5 | 10 |  |
|  | Grade 3 | 8 | 1 | 2 | 3 |  |

| **Anthropometric category** | **Subgroup** | **Not at all** | **A little** | **A lot** | **Very much** | **χ^2^ - DLQI Q7 – Work or study** |
| --- | --- | --- | --- | --- | --- | --- |
| Age | 18-40 | 18 | 2 | 2 | 18 | χ^2^ (114, 6) = 6.712, *p* = 0.348 |
|  | 41-60 | 17 | 3 | 1 | 17 |  |
|  | >60 | 18 | 5 | 4 | 9 |  |
| Residence | Urban | 36 | 4 | 5 | 31 | χ^2^ (114, 3) = 3.593, *p* = 0.309 |
|  | Rural | 17 | 6 | 2 | 13 |  |
| Gender | M | 28 | 3 | 6 | 19 | χ^2^ (114, 3) = 6.126, *p* = 0.106 |
|  | F | 25 | 7 | 1 | 25 |  |
| Localisation | Visible | 35 | 6 | 4 | 22 | χ^2^ (114, 3) = 2.566, *p* = 0.464 |
|  | Occult | 18 | 4 | 3 | 22 |  |
| Marital status | Single | 17 | 2 | 2 | 19 | χ^2^ (114, 3) = 2.607, *p* = 0.456 |
|  | Married | 36 | 8 | 5 | 25 |  |
| Debut | < 5y | 11 | 3 | 1 | 12 | χ^2^ (114, 6) = 1.966, *p* = 0.923 χ^2^ (114, 3) = 1.295, *p* = 0.730, *less than 10 y vs. more than 10 y* |
|  | 5-10 y | 11 | 3 | 2 | 9 |  |
|  | >10 y | 31 | 4 | 4 | 23 |  |
| Level of education | Secondary school | 36 | 9 | 5 | 29 | χ^2^ (114, 3) = 2.314, *p* = 0.510 |
|  | BSc | 17 | 1 | 2 | 15 |  |
| Affected surface | Grade 1 | 22 | 6 | 5 | 21 | χ^2^ (100, 3) = 2.786, *p* = 0.426, *Grade 1 vs. Grade 2* χ^2^ (114, 3) = 2.997, *p* = 0.392*, Grade 1 vs. Grade 2 and 3* |
|  | Grade 2 | 23 | 2 | 2 | 19 |  |
|  | Grade 3 | 8 | 2 | 0 | 4 |  |
| **Anthropometric category** | **Subgroup** | **Not at all** | **A little** | **A lot** | **Very much** | **χ^2^ - DLQI Q8 – Interpersonal relationships** |
| Age | 18-40 | 12 | 8 | 4 | 16 | χ^2^ (114, 6) = 5.094, *p* = 0.532 |
|  | 41-60 | 17 | 6 | 7 | 8 |  |
|  | >60 | 14 | 7 | 6 | 9 |  |
| Residence | Urban | 29 | 13 | 10 | 24 | χ^2^ (114, 3) = 1.242, *p* = 0.743 |
|  | Rural | 14 | 8 | 7 | 9 |  |
| Gender | M | 24 | 10 | 5 | 17 | χ^2^ (114, 3) = 3.508, *p* = 0.320 |
|  | F | 19 | 11 | 12 | 16 |  |
| Localisation | Visible | 24 | 11 | 11 | 21 | χ^2^ (114, 3) = 1.079, *p* = 0.782 |
|  | Occult | 19 | 10 | 6 | 12 |  |
| Marital status | Single | 11 | 9 | 3 | 17 | **χ^2^ (114, 3) = 8.443, *p* = 0.038** Fischer's exact test, *p* = 0.429 |
|  | Married | 32 | 12 | 14 | 16 |  |
| Debut | < 5y | 10 | 5 | 3 | 9 | χ^2^ (114, 6) = 3.280, *p* = 0.773 χ^2^ (114, 3) = 2.942, *p* = 0.401, *less than 10 y vs. more than 10 y* |
|  | 5-10 y | 8 | 4 | 3 | 10 |  |
|  | >10 y | 25 | 12 | 11 | 14 |  |
| Level of education | Secondary school | 30 | 12 | 14 | 23 | χ^2^ (114, 3) = 2.827, *p* = 0.419 |
|  | BSc | 13 | 9 | 3 | 10 |  |
| Affected surface | Grade 1 | 18 | 12 | 5 | 19 | χ^2^ (100, 3) = 5.503, *p* = 0.138, *Grade 1 vs. Grade 2* χ^2^ (114, 3) = 4.906, *p* = 0.179*, Grade 1 vs. Grade 2 and 3* |
|  | Grade 2 | 22 | 5 | 8 | 11 |  |
|  | Grade 3 | 3 | 4 | 4 | 3 |  |

| **Anthropometric category** | **Subgroup** | **Not at all** | **A little** | **A lot** | **Very much** | **χ^2^ - DLQI Q9 – Sexual activity** |
| --- | --- | --- | --- | --- | --- | --- |
| Age | 18-40 | 13 | 3 | 4 | 20 | **χ^2^ (114, 6) = 12.698, *p* = 0.048** |
|  | 41-60 | 15 | 1 | 4 | 18 |  |
|  | >60 | 21 | 5 | 4 | 6 |  |
| Residence | Urban | 32 | 7 | 8 | 29 | χ^2^ (114, 3) = 0.5522, *p* = 0.907 |
|  | Rural | 17 | 2 | 4 | 15 |  |
| Gender | M | 24 | 3 | 5 | 24 | χ^2^ (114, 3) = 1.683, *p* = 0.641 |
|  | F | 25 | 6 | 7 | 20 |  |
| Localisation | Visible | 29 | 3 | 4 | 31 | **χ^2^ (114, 3) = 8.090, *p* = 0.044** Fischer's exact test, *p* = 0.452 |
|  | Occult | 20 | 6 | 8 | 13 |  |
| Marital status | Single | 13 | 3 | 4 | 20 | χ^2^ (114, 3) = 3.680, *p* = 0.298 |
|  | Married | 36 | 6 | 8 | 24 |  |
| Debut | < 5y | 14 | 0 | 0 | 13 | χ^2^ (114, 6) = 11.393, *p* = 0.077 χ^2^ (114, 3) = 5.215, *p* = 0.157, *less than 10 y vs. more than 10 y* |
|  | 5-10 y | 9 | 1 | 5 | 10 |  |
|  | >10 y | 26 | 8 | 7 | 21 |  |
| Level of education | Secondary school | 39 | 5 | 10 | 25 | χ^2^ (114, 3) = 7.571, *p* = 0.056 |
|  | BSc | 10 | 4 | 2 | 19 |  |
| Affected surface | Grade 1 | 23 | 3 | 6 | 22 | χ^2^ (100, 3) = 0.924, *p* = 0.820, *Grade 1 vs. Grade 2* χ^2^ (114, 3) = 0.870, *p* = 0.833*, Grade 1 vs. Grade 2 and 3* |
|  | Grade 2 | 21 | 4 | 6 | 15 |  |
|  | Grade 3 | 5 | 2 | 0 | 7 |  |
| **Anthropometric category** | **Subgroup** | **Not at all** | **A little** | **A lot** | **Very much** | **χ^2^ - DLQI Q10 – Treatment** |
| Age | 18-40 | 20 | 3 | 9 | 8 | χ^2^ (114, 6) = 7.862, *p* = 0.248 |
|  | 41-60 | 20 | 6 | 2 | 10 |  |
|  | >60 | 22 | 4 | 6 | 4 |  |
| Residence | Urban | 40 | 7 | 14 | 15 | χ^2^ (114, 3) = 2.996, *p* = 0.392 |
|  | Rural | 22 | 6 | 3 | 7 |  |
| Gender | M | 35 | 4 | 9 | 8 | χ^2^ (114, 3) = 4.617, *p* = 0.202 |
|  | F | 27 | 9 | 8 | 14 |  |
| Localisation | Visible | 36 | 5 | 11 | 15 | χ^2^ (114, 3) = 3.277, *p* = 0.351 |
|  | Occult | 26 | 8 | 6 | 7 |  |
| Marital status | Single | 21 | 2 | 9 | 8 | χ^2^ (114, 3) = 4.651, *p* = 0.199 |
|  | Married | 41 | 11 | 8 | 14 |  |
| Debut | < 5y | 13 | 3 | 6 | 5 | χ^2^ (114, 6) = 1.932, *p* = 0.926 χ^2^ (114, 3) = 1.536, *p* = 0.674, *less than 10 y vs. more than 10 y* |
|  | 5-10 y | 13 | 3 | 4 | 5 |  |
|  | >10 y | 36 | 7 | 7 | 12 |  |
| Level of education | Secondary school | 48 | 9 | 10 | 12 | χ^2^ (114, 3) = 5.049, *p* = 0.168 |
|  | BSc | 14 | 4 | 7 | 10 |  |
| Affected surface | Grade 1 | 32 | 2 | 12 | 8 | **χ^2^ (100, 3) = 10.291, *p* = 0.016, *Grade 1 vs. Grade 2* χ^2^ (114, 3) = 10.527, *p* = 0.015*, Grade 1 vs. Grade 2 and 3*** |
|  | Grade 2 | 23 | 10 | 4 | 9 |  |
|  | Grade 3 | 7 | 1 | 1 | 5 |  |
