# Supplementary material for: Comprehensive analysis of life quality of patients with vitiligo in Romania: insights from a multivariate approach
Source: Front Med (Lausanne). 2025 May 26;12:1613083. doi: 10.3389/fmed.2025.1613083 (PMC12146156; doi:10.3389/fmed.2025.1613083)
Supplement: Supplementary file 2 [file Table_2.docx]

**Supplementary Table 2.** Statistical analysis of the responses given by the respondents to the items of RSE questionnaire based on anthropometric variables

| **Anthropometric category** | **Subgroup** | **Strongly disagree** | **Disagree** | **Agree** | **Strongly agree** | **χ^2^ - RSE Q1** |
| --- | --- | --- | --- | --- | --- | --- |
| Age | 18-40 | 8 | 10 | 9 | 13 | χ^2^ (114, 6) = 3.683, *p* = 0.720 |
|  | 41-60 | 6 | 8 | 11 | 13 |  |
|  | >60 | 3 | 6 | 12 | 15 |  |
| Residence | Urban | 10 | 15 | 24 | 27 | χ^2^ (114, 3) = 1.670, *p* = 0.644 |
|  | Rural | 7 | 9 | 8 | 14 |  |
| Gender | M | 8 | 13 | 16 | 19 | χ^2^ (114, 3) = 0.4100, *p* = 0.938 |
|  | F | 9 | 11 | 16 | 22 |  |
| Localisation | Visible | 13 | 13 | 18 | 23 | χ^2^ (114, 3) = 2.613, *p* = 0.455 |
|  | Occult | 4 | 11 | 14 | 18 |  |
| Marital status | Single | 7 | 14 | 6 | 13 | **χ^2^ (114, 3) = 9.926, *p* = 0.019 Fischer's exact test, *p* = 0.0084** |
|  | Married | 10 | 10 | 26 | 28 |  |
| Debut | < 5y | 5 | 6 | 8 | 8 | χ2 (114, 6) = 1.868, *p* = 0.931 χ^2^ (114, 3) = 1.526, *p* = 0.676, *less than 10 y vs. more than 10 y* |
|  | 5-10 y | 5 | 5 | 6 | 9 |  |
|  | >10 y | 7 | 13 | 18 | 24 |  |
| Level of education | Secondary school | 11 | 16 | 20 | 32 | χ^2^ (114, 3) = 2.417, *p* = 0.490 |
|  | BSc | 6 | 8 | 12 | 9 |  |
| Affected surface | Grade 1 | 7 | 14 | 14 | 19 | χ^2^ (100, 3) = 0.821, *p* = 0.845, *Grade 1 vs. Grade 2* χ^2^ (114, 3) = 1.604, *p* = 0.658*, Grade 1 vs. Grade 2 and 3* |
|  | Grade 2 | 6 | 9 | 15 | 16 |  |
|  | Grade 3 | 4 | 1 | 3 | 6 |  |
| **Anthropometric category** | **Subgroup** | **Strongly disagree** | **Disagree** | **Agree** | **Strongly agree** | **χ^2^ - RSE Q2** |
| Age | 18-40 | 16 | 11 | 10 | 3 | χ^2^ (114, 6) = 1.873, *p* = 0.931 |
|  | 41-60 | 11 | 14 | 10 | 3 |  |
|  | >60 | 10 | 12 | 11 | 3 |  |
| Residence | Urban | 25 | 22 | 24 | 5 | χ^2^ (114, 3) = 2.991, *p* = 0.393 |
|  | Rural | 12 | 15 | 7 | 4 |  |
| Gender | M | 17 | 18 | 16 | 5 | χ^2^ (114, 3) = 0.379, *p* = 0.945 |
|  | F | 20 | 19 | 15 | 4 |  |
| Localisation | Visible | 22 | 21 | 18 | 6 | χ^2^ (114, 3) = 0.307, *p* = 0.959 |
|  | Occult | 15 | 16 | 13 | 3 |  |
| Marital status | Single | 14 | 12 | 11 | 3 | χ^2^ (114, 3) = 0.2517, *p* = 0.969 |
|  | Married | 23 | 25 | 20 | 6 |  |
| Debut | < 5y | 8 | 9 | 8 | 2 | χ^2^ (114, 6) = 3.769, *p* = 0.708 χ^2^ (114, 3) = 2.172, *p* = 0.537, *less than 10 y vs. more than 10 y* |
|  | 5-10 y | 8 | 6 | 7 | 4 |  |
|  | >10 y | 21 | 22 | 16 | 3 |  |
| Level of education | Secondary school | 27 | 26 | 18 | 8 | χ^2^ (114, 3) = 3.714, *p* = 0.294 |
|  | BSc | 10 | 11 | 13 | 1 |  |
| Affected surface | Grade 1 | 18 | 16 | 13 | 7 | χ^2^ (100, 3) = 4.190, *p* = 0.242, *Grade 1 vs. Grade 2* χ^2^ (114, 3) = 3.982, *p* = 0.263*, Grade 1 vs. Grade 2 and 3* |
|  | Grade 2 | 15 | 17 | 13 | 1 |  |
|  | Grade 3 | 4 | 4 | 5 | 1 |  |

| **Anthropometric category** | **Subgroup** | **Strongly disagree** | **Disagree** | **Agree** | **Strongly agree** | **χ^2^ - RSE Q3** |
| --- | --- | --- | --- | --- | --- | --- |
| Age | 18-40 | 17 | 11 | 5 | 7 | χ^2^ (114, 6) = 4.487, *p* = 0.611 |
|  | 41-60 | 15 | 8 | 9 | 6 |  |
|  | >60 | 10 | 9 | 11 | 6 |  |
| Residence | Urban | 26 | 23 | 15 | 12 | χ^2^ (114, 3) = 4.052, *p* = 0.256 |
|  | Rural | 16 | 5 | 10 | 7 |  |
| Gender | M | 18 | 14 | 10 | 14 | χ^2^ (114, 3) = 6.087, *p* = 0.107 |
|  | F | 24 | 14 | 15 | 5 |  |
| Localisation | Visible | 27 | 17 | 14 | 9 | χ^2^ (114, 3) = 1.670, *p* = 0.644 |
|  | Occult | 15 | 11 | 11 | 10 |  |
| Marital status | Single | 17 | 11 | 6 | 6 | χ^2^ (114, 3) = 2.204, *p* = 0.531 |
|  | Married | 25 | 17 | 19 | 13 |  |
| Debut | < 5y | 9 | 8 | 2 | 8 | χ2 (114, 6) = 9.222, *p* = 0.162 χ^2^ (114, 3) = 4.615, *p* = 0.202, *less than 10 y vs. more than 10 y* |
|  | 5-10 y | 12 | 5 | 5 | 3 |  |
|  | >10 y | 21 | 15 | 18 | 8 |  |
| Level of education | Secondary school | 30 | 16 | 19 | 14 | χ^2^ (114, 3) = 2.734, *p* = 0.435 |
|  | BSc | 12 | 12 | 6 | 5 |  |
| Affected surface | Grade 1 | 23 | 13 | 9 | 9 | χ^2^ (100, 3) = 1.352, *p* = 0.717, *Grade 1 vs. Grade 2* χ^2^ (114, 3) = 2.227, *p* = 0.527*, Grade 1 vs. Grade 2 and 3* |
|  | Grade 2 | 15 | 12 | 11 | 8 |  |
|  | Grade 3 | 4 | 3 | 5 | 2 |  |
| **Anthropometric category** | **Subgroup** | **Strongly disagree** | **Disagree** | **Agree** | **Strongly agree** | **χ^2^ - RSE Q4** |
| Age | 18-40 | 6 | 8 | 17 | 9 | χ^2^ (114, 6) = 11.449, *p* = 0.075 |
|  | 41-60 | 2 | 13 | 12 | 11 |  |
|  | >60 | 2 | 4 | 22 | 8 |  |
| Residence | Urban | 10 | 15 | 32 | 19 | χ^2^ (114, 3) = 5.871, *p* = 0.118 |
|  | Rural | 0 | 10 | 19 | 9 |  |
| Gender | M | 5 | 10 | 26 | 15 | χ^2^ (114, 3) = 1.128, *p* = 0.770 |
|  | F | 5 | 15 | 25 | 13 |  |
| Localisation | Visible | 6 | 13 | 32 | 16 | χ^2^ (114, 3) = 0.8423, *p* = 0.839 |
|  | Occult | 4 | 12 | 19 | 12 |  |
| Marital status | Single | 5 | 10 | 17 | 8 | χ^2^ (114, 3) = 1.832, *p* = 0.608 |
|  | Married | 5 | 15 | 34 | 20 |  |
| Debut | < 5y | 4 | 7 | 11 | 5 | χ^2^ (114, 6) = 3.842, *p* = 0.698 χ^2^ (114, 3) = 2.635, *p* = 0.451, *less than 10 y vs. more than 10 y* |
|  | 5-10 y | 3 | 4 | 11 | 7 |  |
|  | >10 y | 3 | 14 | 29 | 16 |  |
| Level of education | Secondary school | 7 | 18 | 34 | 20 | χ^2^ (114, 3) = 0.3138, *p* = 0.957 |
|  | BSc | 3 | 7 | 17 | 8 |  |
| Affected surface | Grade 1 | 5 | 10 | 27 | 12 | χ^2^ (100, 3) = 0.3344, *p* = 0.953, *Grade 1 vs. Grade 2* χ^2^ (114, 3) = 1.436, *p* = 0.697*, Grade 1 vs. Grade 2 and 3* |
|  | Grade 2 | 5 | 8 | 21 | 12 |  |
|  | Grade 3 | 0 | 7 | 3 | 4 |  |

| **Anthropometric category** | **Subgroup** | **Strongly disagree** | **Disagree** | **Agree** | **Strongly agree** | **χ^2^ - RSE Q5** |
| --- | --- | --- | --- | --- | --- | --- |
| Age | 18-40 | 6 | 8 | 13 | 13 | χ^2^ (114, 6) = 12.141, *p* = 0.059 |
|  | 41-60 | 9 | 5 | 10 | 14 |  |
|  | >60 | 8 | 15 | 4 | 9 |  |
| Residence | Urban | 16 | 16 | 20 | 24 | χ^2^ (114, 3) = 1.896, *p* = 0.594 |
|  | Rural | 7 | 12 | 7 | 12 |  |
| Gender | M | 11 | 20 | 12 | 13 | **χ^2^ (114, 3) = 8.265, *p* = 0.041 Fischer's exact test, *p* = 0.038** |
|  | F | 12 | 8 | 15 | 23 |  |
| Localisation | Visible | 12 | 14 | 17 | 24 | χ^2^ (114, 3) = 2.424, *p* = 0.489 |
|  | Occult | 11 | 14 | 10 | 12 |  |
| Marital status | Single | 6 | 10 | 11 | 13 | χ^2^ (114, 3) = 1.218, *p* = 0.747 |
|  | Married | 17 | 18 | 16 | 23 |  |
| Debut | < 5y | 5 | 6 | 7 | 9 | χ^2^ (114, 6) = 2.385, *p* = 0.881 χ^2^ (114, 3) = 1.963, *p* = 0.580, *less than 10 y vs. more than 10 y* |
|  | 5-10 y | 5 | 4 | 6 | 10 |  |
|  | >10 y | 13 | 18 | 14 | 17 |  |
| Level of education | Secondary school | 15 | 19 | 21 | 24 | χ^2^ (114, 3) = 1.237, *p* = 0.744 |
|  | BSc | 8 | 9 | 6 | 12 |  |
| Affected surface | Grade 1 | 10 | 14 | 13 | 17 | χ^2^ (100, 3) = 0.3079, *p* = 0.959, *Grade 1 vs. Grade 2* χ^2^ (114, 3) = 0.224, *p* = 0.974*, Grade 1 vs. Grade 2 and 3* |
|  | Grade 2 | 9 | 11 | 13 | 13 |  |
|  | Grade 3 | 4 | 3 | 1 | 6 |  |
| **Anthropometric category** | **Subgroup** | **Strongly disagree** | **Disagree** | **Agree** | **Strongly agree** | **χ^2^ - RSE Q6** |
| Age | 18-40 | 6 | 12 | 9 | 13 | χ^2^ (114, 6) = 10.522, *p* = 0.104 |
|  | 41-60 | 9 | 8 | 15 | 6 |  |
|  | >60 | 7 | 16 | 8 | 5 |  |
| Residence | Urban | 16 | 23 | 22 | 15 | χ^2^ (114, 3) = 0.7386, *p* = 0.864 |
|  | Rural | 6 | 13 | 10 | 9 |  |
| Gender | M | 14 | 12 | 14 | 16 | χ^2^ (114, 3) = 8.771, *p* = 0.033 Fischer's exact test, *p* = 0.454 |
|  | F | 8 | 24 | 18 | 8 |  |
| Localisation | Visible | 13 | 20 | 21 | 13 | χ^2^ (114, 3) = 0.985, *p* = 0.805 |
|  | Occult | 9 | 16 | 11 | 11 |  |
| Marital status | Single | 6 | 12 | 11 | 11 | χ^2^ (114, 3) = 1.862, *p* = 0.601 |
|  | Married | 16 | 24 | 21 | 13 |  |
| Debut | < 5y | 5 | 8 | 7 | 7 | χ^2^ (114, 6) = 1.127, *p* = 0.980 χ^2^ (114, 3) = 1.049, *p* = 0.789, *less than 10 y vs. more than 10 y* |
|  | 5-10 y | 5 | 8 | 6 | 6 |  |
|  | >10 y | 12 | 20 | 19 | 11 |  |
| Level of education | Secondary school | 13 | 28 | 23 | 15 | χ^2^ (114, 3) = 2.915, *p* = 0.405 |
|  | BSc | 9 | 8 | 9 | 9 |  |
| Affected surface | Grade 1 | 11 | 17 | 16 | 10 | χ^2^ (100, 3) = 0.228, *p* = 0.928, *Grade 1 vs. Grade 2* χ^2^ (114, 3) = 0.463, *p* = 0.927*, Grade 1 vs. Grade 2 and 3* |
|  | Grade 2 | 8 | 16 | 13 | 9 |  |
|  | Grade 3 | 3 | 3 | 3 | 5 |  |

| **Anthropometric category** | **Subgroup** | **Strongly disagree** | **Disagree** | **Agree** | **Strongly agree** | **χ^2^ - RSE Q7** |
| --- | --- | --- | --- | --- | --- | --- |
| Age | 18-40 | 14 | 7 | 14 | 5 | χ^2^ (114, 6) = 6.101, *p* = 0.412 |
|  | 41-60 | 7 | 12 | 12 | 7 |  |
|  | >60 | 6 | 10 | 12 | 8 |  |
| Residence | Urban | 21 | 19 | 21 | 15 | χ^2^ (114, 3) = 4.366, *p* = 0.225 |
|  | Rural | 6 | 10 | 17 | 5 |  |
| Gender | M | 13 | 15 | 14 | 14 | χ^2^ (114, 3) = 5.870, *p* = 0.118 |
|  | F | 14 | 14 | 24 | 6 |  |
| Localisation | Visible | 17 | 15 | 22 | 13 | χ^2^ (114, 3) = 1.122, *p* = 0.772 |
|  | Occult | 10 | 14 | 16 | 7 |  |
| Marital status | Single | 13 | 8 | 12 | 7 | χ^2^ (114, 3) = 2.944, *p* = 0.400 |
|  | Married | 14 | 21 | 26 | 13 |  |
| Debut | < 5y | 6 | 9 | 8 | 4 | χ^2^ (114, 6) = 9.911, *p* = 0.128 χ^2^ (114, 3) = 4.667, *p* = 0.198, *less than 10 y vs. more than 10 y* |
|  | 5-10 y | 11 | 3 | 6 | 5 |  |
|  | >10 y | 10 | 17 | 24 | 11 |  |
| Level of education | Elementary | 1 | 1 | 2 | 1 | χ^2^ (114, 3) = 1.782, *p* = 0.619, *BSC vs. undergraduate studies* |
|  | Secondary school | 17 | 19 | 27 | 11 |  |
|  | BSc | 9 | 9 | 9 | 8 |  |
| Affected surface | Grade 1 | 17 | 12 | 16 | 9 | χ^2^ (100, 3) = 1.596, *p* = 0.660, *Grade 1 vs. Grade 2* χ^2^ (114, 3) = 3.518, *p* = 0.318*, Grade 1 vs. Grade 2 and 3* |
|  | Grade 2 | 10 | 11 | 18 | 7 |  |
|  | Grade 3 | 0 | 6 | 4 | 4 |  |
| **Anthropometric category** | **Subgroup** | **Strongly disagree** | **Disagree** | **Agree** | **Strongly agree** | **χ^2^ - RSE Q8** |
| Age | 18-40 | 3 | 8 | 7 | 22 | χ^2^ (114, 6) = 5.479, *p* = 0.484 |
|  | 41-60 | 2 | 5 | 11 | 20 |  |
|  | >60 | 5 | 5 | 12 | 14 |  |
| Residence | Urban | 8 | 14 | 17 | 37 | χ^2^ (114, 3) = 3.159, *p* = 0.368 |
|  | Rural | 2 | 4 | 13 | 19 |  |
| Gender | M | 8 | 7 | 14 | 27 | χ^2^ (114, 3) = 4.660, *p* = 0.198 |
|  | F | 2 | 11 | 16 | 29 |  |
| Localisation | Visible | 5 | 8 | 20 | 34 | χ^2^ (114, 3) = 2.701, *p* = 0.440 |
|  | Occult | 5 | 10 | 10 | 22 |  |
| Marital status | Single | 4 | 6 | 7 | 23 | χ^2^ (114, 3) = 2.830, *p* = 0.418 |
|  | Married | 6 | 12 | 23 | 33 |  |
| Debut | < 5y | 1 | 7 | 5 | 14 | χ^2^ (114, 6) = 9.898, *p* = 0.129 χ^2^ (114, 3) = 6.596, *p* = 0.086, *less than 10 y vs. more than 10 y* |
|  | 5-10 y | 4 | 4 | 3 | 14 |  |
|  | >10 y | 5 | 7 | 22 | 28 |  |
| Level of education | Elementary | 2 | 0 | 1 | 2 | χ^2^ (114, 3) = 1.636, *p* = 0.651, *BSC vs. undergraduate studies* |
|  | Secondary school | 4 | 14 | 18 | 38 |  |
|  | BSc | 4 | 4 | 11 | 16 |  |
| Affected surface | Grade 1 | 3 | 10 | 9 | 32 | χ^2^ (100, 3) = 4.512, *p* = 0.211, *Grade 1 vs. Grade 2* χ^2^ (114, 3) = 7.47, *p* = 0.058*, Grade 1 vs. Grade 2 and 3* |
|  | Grade 2 | 5 | 8 | 14 | 19 |  |
|  | Grade 3 | 2 | 0 | 7 | 5 |  |

| **Anthropometric category** | **Subgroup** | **Strongly disagree** | **Disagree** | **Agree** | **Strongly agree** | **χ^2^ - RSE Q9** |
| --- | --- | --- | --- | --- | --- | --- |
| Age | 18-40 | 3 | 6 | 11 | 20 | χ^2^ (114, 6) = 4.431, *p* = 0.618 |
|  | 41-60 | 7 | 8 | 6 | 17 |  |
|  | >60 | 4 | 9 | 8 | 15 |  |
| Residence | Urban | 8 | 19 | 17 | 32 | χ^2^ (114, 3) = 3.837, *p* = 0.280 |
|  | Rural | 6 | 4 | 8 | 20 |  |
| Gender | M | 8 | 10 | 14 | 24 | χ^2^ (114, 3) = 1.310, *p* = 0.727 |
|  | F | 6 | 13 | 11 | 28 |  |
| Localisation | Visible | 7 | 16 | 13 | 31 | χ^2^ (114, 3) = 2.039, *p* = 0.564 |
|  | Occult | 7 | 7 | 12 | 21 |  |
| Marital status | Single | 5 | 7 | 7 | 21 | χ^2^ (114, 3) = 1.413, *p* = 0.702 |
|  | Married | 9 | 16 | 18 | 31 |  |
| Debut | < 5y | 5 | 2 | 9 | 11 | χ^2^ (114, 6) = 6.467, *p* = 0.373 χ^2^ (114, 3) = 2.323, *p* = 0.508, *less than 10 y vs. more than 10 y* |
|  | 5-10 y | 2 | 6 | 5 | 12 |  |
|  | >10 y | 7 | 15 | 11 | 29 |  |
| Level of education | Elementary | 2 | 2 | 0 | 1 | χ^2^ (114, 3) = 4.171, *p* = 0.543, *BSC vs. undergraduate studies* |
|  | Secondary school | 9 | 10 | 18 | 37 |  |
|  | BSc | 3 | 11 | 7 | 14 |  |
| Affected surface | Grade 1 | 8 | 8 | 9 | 29 | χ^2^ (100, 3) = 5.805, *p* = 0.121, *Grade 1 vs. Grade 2* χ^2^ (114, 3) = 4.766, *p* = 0.190*, Grade 1 vs. Grade 2 and 3* |
|  | Grade 2 | 3 | 11 | 14 | 18 |  |
|  | Grade 3 | 3 | 4 | 2 | 5 |  |
| **Anthropometric category** | **Subgroup** | **Strongly disagree** | **Disagree** | **Agree** | **Strongly agree** | **χ^2^ - RSE Q10** |
| Age | 18-40 | 26 | 6 | 3 | 5 | χ^2^ (114, 6) = 3.109, *p* = 0.795 |
|  | 41-60 | 20 | 9 | 4 | 5 |  |
|  | >60 | 18 | 7 | 6 | 5 |  |
| Residence | Urban | 42 | 16 | 8 | 10 | χ^2^ (114, 3) = 0.5487, *p* = 0.908 |
|  | Rural | 22 | 6 | 5 | 5 |  |
| Gender | M | 32 | 8 | 8 | 8 | χ^2^ (114, 3) = 2.361, *p* = 0.500 |
|  | F | 32 | 14 | 5 | 7 |  |
| Localisation | Visible | 39 | 12 | 8 | 8 | χ^2^ (114, 3) = 0.5102, *p* = 0.916 |
|  | Occult | 25 | 10 | 5 | 7 |  |
| Marital status | Single | 25 | 5 | 3 | 7 | χ^2^ (114, 3) = 3.626, *p* = 0.305 |
|  | Married | 39 | 17 | 10 | 8 |  |
| Debut | < 5y | 15 | 5 | 3 | 4 | χ^2^ (114, 6) = 0.760, *p* = 0.993 χ^2^ (114, 3) = 0.233, *p* = 0.972, *less than 10 y vs. more than 10 y* |
|  | 5-10 y | 15 | 5 | 3 | 2 |  |
|  | >10 y | 34 | 12 | 7 | 9 |  |
| Level of education | Secondary school | 46 | 16 | 7 | 10 | χ^2^ (114, 3) = 1.829, *p* = 0.609 |
|  | BSc | 18 | 6 | 6 | 5 |  |
| Affected surface | Grade 1 | 35 | 7 | 5 | 7 | χ^2^ (100, 3) = 3.256, *p* = 0.354, *Grade 1 vs. Grade 2* χ^2^ (114, 3) = 3.926, *p* = 0.270*, Grade 1 vs. Grade 2 and 3* |
|  | Grade 2 | 23 | 12 | 5 | 6 |  |
|  | Grade 3 | 6 | 3 | 3 | 2 |  |
