# Supplementary material for: Comprehensive analysis of life quality of patients with vitiligo in Romania: insights from a multivariate approach
Source: Front Med (Lausanne). 2025 May 26;12:1613083. doi: 10.3389/fmed.2025.1613083 (PMC12146156; doi:10.3389/fmed.2025.1613083)
Supplement: Supplementary file 3 [file Table_3.docx]

**Supplementary Table 3**. Correlation matrix of the responses given to the DLQI and RSE questionnaires

|  | **RSE Q1** | **RSE Q2** | **RSE Q3** | **RSE Q4** | **RSE Q5** | **RSE Q6** | **RSE Q7** | **RSE Q8** | **RSE Q9** | **RSE Q10** | **Total RSE score** |
| --- | --- | --- | --- | --- | --- | --- | --- | --- | --- | --- | --- |
| **DLQI Q1** | -0.30 | -0.35 | -0.25 | -0.29 | -0.24 | -0.22 | -0.39 | -0.13 | -0.14 | -0.13 |  |
| **DLQI Q2** | -0.36 | -0.47 | -0.18 | -0.41 | -0.20 | -0.27 | -0.56 | -0.26 | -0.24 | -0.22 |  |
| **DLQI Q3** | -0.50 | -0.35 | -0.21 | -0.38 | -0.28 | -0.47 | -0.46 | -0.42 | -0.27 | -0.30 |  |
| **DLQI Q4** | -0.42 | -0.4 | -0.23 | -0.42 | -0.18 | -0.49 | -0.52 | -0.33 | -0.32 | -0.33 |  |
| **DLQI Q5** | -0.44 | -0.43 | -0.19 | -0.2 | -0.07 | -0.44 | -0.51 | -0.25 | -0.29 | -0.32 |  |
| **DLQI Q6** | -0.48 | -0.39 | -0.32 | -0.42 | -0.25 | -0.41 | -0.56 | -0.37 | -0.41 | -0.38 |  |
| **DLQI Q7** | -0.46 | -0.42 | -0.28 | -0.42 | -0.22 | -0.27 | -0.55 | -0.30 | -0.33 | -0.34 |  |
| **DLQI Q8** | -0.40 | -0.46 | -0.21 | -0.32 | -0.32 | -0.25 | -0.48 | -0.38 | -0.35 | -0.36 |  |
| **DLQI Q9** | -0.54 | -0.42 | -0.22 | -0.27 | -0.26 | -0.46 | -0.50 | -0.45 | -0.25 | -0.36 |  |
| **DLQI Q10** | -0.38 | -0.31 | -0.24 | -0.33 | -0.37 | -0.34 | -0.40 | -0.29 | -0.32 | -0.21 |  |
| **Total DLQI score** | | | | | | | | | | | -0.70 |
